# Healthy Houses

**Published:** 1874-11

**Authors:** 


### HEALTHY HOUSES.

Most persons in selecting a town site prefer a level plain, yet all know that the mountain is healthier than the valley. An elevated situation is less liable to disease than a flat ; the difference in salubrity is less, from the fact that water tends downwards, leaving a dry soil ; dampness and disease go hand-in-hand.

There is another reason. In flat localities all the accumulations of dirt and filth on the surface are rained upon, are dissolved, and the water is saturated with them. This water sinks into the ground a few feet, and there remains. It is this water which supplies the wells, which is mixed with our food and swallowed into our stomachs by the gallon every day. It is not any wonder that the inhabitants of

Europe have discarded the use of water entirely as a drink. To have pure water, the source of it should be higher than the town, and if in the country, higher than any of the dwellings in the neighborhood; and yet, in most cases, the "spring" or the well has to be gone down to, is almost always at the bottom of the hill, or other elevation. In respect to the selection of a site for a family residence, especially in cities, it should either be on a sandy soil or on a natural hill, never over a filling. As this must be done, there should be a system of drain-pipes to carry the water abundantly and rapidly away. Many a family mansion has been built with the accumulations of the savings of half a life-time, to make the graves of half the household in a few months, from neglect of the precautions for thorough draining and a proper water supply for drinking and cooking.

In a ward in Brooklyn, with a population of about ten thousand, with natural drainage, elevation, and a sandy soil besides, only one and a half persons in the thousand died of consumption; in the adjoining ward, more than three times as many for each thousand; more than three times as many died of consumption who were living for the most part on flat land, on soil saturated with dampness.

In a city ward, elevated and well drained, two and a half persons out of each thousand died of zymotic diseases—that is, of maladies arising from bad air and bad water, from uncleanliness generally. In two other wards built mainly on flat land, some of it filled in, four times as many died of zymotic diseases—that is, ten out of each thousand. These are facts which should be kept before the mind of all who select new houses, or localities for new houses, in which they expect to reside with their families as long as they can. The neglect of these things has given rise to the observation very generally made: "He built a house only to move into it and die."

